# Supporting Mothers of Very Preterm Infants and Breast Milk Production: A Review of the Role of Galactogogues

**DOI:** 10.3390/nu10050600

**Published:** 2018-05-12

**Authors:** Elizabeth V. Asztalos

**Affiliations:** Department of Newborn and Developmental Paediatrics, Sunnybrook Health Sciences Centre, University of Toronto, M4N 3M5 Toronto, ON, Canada; elizabeth.asztalos@sunnybrook.ca; Tel.: +1-416-480-6100 (ext. 87791); Fax: +1-416-480-5612

**Keywords:** breast milk, galactogogues, mothers of preterm infants

## Abstract

Human milk, either mother’s own milk or donor human milk, is recommended as the primary source of nutrition for very preterm infants. Initiatives should be in place in neonatal units to provide support to the mother as she strives to initiate and maintain a supply of breast milk for her infant. The use of galactogogues are considered when these initiatives alone may not be successful in supporting mothers in this endeavor. Although there are non-pharmacologic compounds, this review will focus on the pharmacologic galactogogues currently available and the literature related to their use in mothers of very preterm infants.

## 1. Introduction

The very preterm infant (<30 weeks gestation) is faced with an array of serious morbidities, which can include sepsis (late-onset), necrotizing enterocolitis (NEC), retinopathy of prematurity, bronchopulmonary dysplasia (BPD), and intracranial white matter injury [1,2,3,4,5,6]. Human milk is the recommended nutritional support for the very preterm infant as it aids in reducing these morbidities and improves the neurodevelopmental outcomes for these infants [7,8,9,10]. The bioactive components found in breast milk are thought to promote gastrointestinal development, provide substrate for brain development and reduce the incidence of sepsis and necrotizing enterocolitis, both of which are linked in part to a negative impact on neurodevelopment [11,12,13,14]. Based on these clinical information, it is recommended that very preterm infants receive breast milk, preferably mother’s own milk, as the primary source of nutrition rather than rely on preterm formula [15]. Consequently, mothers are encouraged to initiate hand expression and pumping within hours of giving birth to provide breast milk for their infants. With very preterm infants requiring hospitalization for anywhere from 10–16 weeks, continued and sustained breast milk volumes can prove to be a challenge to even the most dedicated of mothers. Many mothers of very preterm infants, for a variety of reasons such as illness, stress and other factors related to preterm birth, are unable to exclusively feed their children [16,17,18,19,20,21].

## 2. Breast Milk Production in Mothers of Preterm Infants

Lactogenesis (milk synthesis) is noted to start around mid-pregnancy and has been referred to as having 2 stages (lactogenesis I and II) which are under the influence of hormones, namely estrogen, insulin, cortisol, progesterone, prolactin, and human placental lactogen [22,23,24,25]. Lactogenesis I represents the secretory differentiation phase where the mammary epithelial cells differentiate into secretory mammary epithelial cells with the capacity to synthesize milk constituents such as lactose, total proteins and immunoglobulins. After parturition, the secretory phase of lactogenesis or lactogenesis II is triggered by the rapid decline of serum maternal progesterone that occurs with the expulsion of the placenta; in addition, this leads to a drop in estrogen levels while prolactin levels remain high along with insulin and cortisol [24,25]. Colostrum is produced during the first 4 days postpartum, followed by transitional milk secretion for the next 10 days followed by mature milk production [26]. Milk volume rapidly increases after the first 24 h postpartum and stabilizes after 1 month postpartum to an average volume of 750–800 mL/24 h for the term infant [27,28]. Milk production is increased by efficient and timely removal of milk, with adequate milk removal by day 3 postpartum being critical to the establishment of ongoing successful lactation [29]. Milk production is regulated by endocrine hormones (prolactin and oxytocin) as well as adequate and regular milk removal. Prolactin is required to maintain milk yield while oxytocin is released in response to suckling and induces the contraction of myoepithelial cells surrounding mammary alveoli triggering milk ejection, “milk let-down” [25]. Once milk secretion is established, hormone levels are maintained at low levels and ongoing production is regulated by consistent and regular milk removal (autocrine control); in the term infant, the volume of milk produced is determined by how the breast is emptied at feedings which, in turn, is determined by the infant’s appetite [29,30,31,32].

Preterm birth may alter the normal sequence of lactogenesis. A delay in secretory activation can be associated with a negative impact on successful lactation [33,34]. Mothers of preterm infants can have problems at this stage as a result of their preterm delivery, antenatal corticosteroids, stress, maternal illness and operative delivery [19,20,21]. Mothers of very preterm infants must establish their milk supply through mechanical expression as the normal mechanism of infant suckling is limited in the very preterm infant [29,35].

Studies have emphasized the importance of establishing an adequate milk production in the early postpartum period for mothers of preterm infants. In a study involving 95 mothers from four tertiary care centers in the Midwest United States, the milk volume expressed on day 4 postpartum was found to be predictive of an inadequate milk supply at 6 weeks postpartum. Mothers producing less than 140 mL/day on day 4 were found to be 9.5 times more at risk of low or inadequate milk production by 6 weeks postpartum [36].

Maintaining a milk volume in amounts sufficient to meet the nutritional needs of their very preterm infants can be challenging for many mothers [17,18,37,38]. A volume of 500 mL/day or 3500 mL/week (equivalent to a mother pumping 80–100 mL/pumping, six times a day) has been identified as the minimum milk volume a mother of a preterm infant should pump in order to meet the needs of her infant at discharge [39]. If a mother is producing >3500 mL by week 2, it can be expected that she will produce this ongoing adequate amount in weeks 4 and 5. If a mother is producing ≥1700 mL/week but <3500 mL by week 2, she has approximately a 50% likelihood of reaching the minimum of 3500 mL/week by week 5 postpartum. For a mother who is producing <1700 mL/week (<40 mL/pumping), the outlook is grim with 100% not achieving the goal of 500 mL/day by weeks 4–5 postpartum.

The inadequate milk volume and declining production over the subsequent weeks pose challenges for the mother eager to provide milk for her infant Additional approaches may need to be explored for those mothers who show a decline in production and will likely stop expression of breast milk for their infant.

## 3. Use of Galactogogues for Breast Milk Production—A Review of the Literature

Many non-pharmacological measures have been found to contribute to variable levels of success in augmenting the breast milk production in mothers of preterm infants [38]. While these approaches may be helpful, it is critical to emphasize that the primary effective strategy for optimizing breast milk volume is frequent and effective breast emptying [29]. In the setting of reduced breast milk volume, galactogogues can be added to an increased pumping regime to augment breast milk volume.

Medications which have galactogogue capabilities generally augment lactation by exerting its effects through either oxytocin or prolactin [40,41]. Oxytocin nasal spray has been evaluated in 3 clinical trials, but negative clinical experience and low use led to the spray being discontinued in many countries thereby limiting its use on a widespread nature [41]. Sulpiride is a substituted benzamide antipsychotic medication. It is an antagonist of dopamine that increases serum prolactin levels similar to other galactogogues. It has poor bioavailability (35%) and has many of the same side effects and complications as other antipsychotics including sedation, extrapyramidal effects, tardive dyskinesia, and neuroleptic malignant syndrome making its use less appealing [41].

The primary medications used today for prolactin production are, like sulpiride, dopamine antagonists. They increase serum prolactin by counteracting the inhibitory influence of dopamine on prolactin secretion. The medications studied most widely for their galactogogue capabilities have been metoclopramide and domperidone. Both medications are used in an “off-label” capacity, i.e., they have not been authorized for use in lactation support. In addition, availability of these medications vary; domperidone, in particular, is available in most countries but not in the United States. A search in the common literature databases (Medline, CINAHL, EMBASE, OVID, Cochrane Library) was done to identify studies or trials evaluating these two pharmacologic galactogogues in mothers of preterm infants.

Metoclopromide augments lactation by antagonizing the release of dopamine in the central nervous system. Because the medication exerts its effects centrally, it can cause extrapyramidal side effects which may include tremor, bradykinesia and other dystonic reactions [40,41].

Seventeen studies were identified evaluating metoclopromide to improve breast milk production (Table 1).

Many studies were done well before 2000 and mostly in mothers with term infants. However, three were conducted with mothers of preterm infants, Ehrenkranz et al. [51], Hansen et al. [56], and Fife et al. [58]. Although not a randomized clinical trial (RCT), Ehrenkranz demonstrated an increase in daily breast milk production with metoclopramide from 93.3 ± 18.0 mL/day to 197.4 ± 32.3 mL/day between the first and seventh day of therapy [51]. The other two, Hansen et al. [56] and Fife [58], found no difference in breast milk volume. These two studies had methodological concerns in that all mothers were enrolled without any evaluation of their ability to produce milk. The inclusion of mothers who would not have had any difficulty in breast milk production may have minimized differences between the groups.

Domperidone is a potent dopamine D_2_ receptor antagonist and was developed and marketed as a prokinetic and antiemetic agent. By blocking dopamine D_2_ receptors in the anterior pituitary, domperidone stimulates the release of prolactin. Domperidone is less lipid soluble, has a higher molecular weight and has lower protein binding (>90%) than metoclopromide (40%). These characteristics appear to prevent domperidone from crossing the blood brain barrier and therefore less likely to cause the extra pyramidal effects often seen with metoclopromide [59,60]. This characteristic made domperidone more appealing in use compared to metoclopramide. In addition, early studies in the 1980’s evaluating its efficacy in augmenting breast milk production [50,51] made this medication more enticing to consider, particularly in mothers of preterm infants.

Nine studies involving domperidone are outlined in Table 2. Seven of these studies were conducted in mothers of preterm infants. All of the studies were small in terms of number of mothers enrolled.

da Silva et al. was the first RCT to evaluate the efficacy of domperidone in mothers of preterm infants [63]. In this study, there was a mean increase in breast milk yield from days 2 to 7 in the domperidone group (49.5 mL, standard deviation 29.4 mL) compared to the placebo group (8.0 mL, standard deviation 39.5 mL) (*p* <0.05) as well as an increase in serum prolactin (*p* = 0.008). Wan et al. evaluated a dose-response relationship between 30 and 60 mg daily [64]. Serum prolactin increased for both doses but was not dose-dependent. In addition, only two-thirds of the mothers (4 out of 6) were identified as “responders” and showed a significant increase in milk production which was also dose-dependent. Campbell-Yeo et al. randomized 46 mothers to either domperidone 10 mg three times daily or placebo equivalent for 14 days [65]. Although the study’s primary goal was to evaluate the effect of domperidone on the nutrient composition of preterm human milk compared to those mothers having received a placebo, there was a significant increase in serum prolactin (*p* = 0.07) and breast milk volumes (*p* = 0.005) in the domperidone group. The mean within-subject increase by day 14 was 267% in the domperidone group (184 to 380 mL) compared to 19% in placebo group (218 to 250 mL). This trial did suggest that a larger yield in breast milk production could be achieved with the additional week as compared to the earlier trial.

Ingram et al. compared the effects of domperidone and metoclopramide on breast milk output in mothers of preterm infants and found no significant differences between the two galactogogues [66]. Both groups showed an increase in breast milk volume. Mothers in the domperidone group achieved a mean of 96.3% in milk volume compared to 93.7% increase for metoclopramide.

Knoppert et al. enrolled 12 mothers between 14–21 days post-delivery to evaluate the effectiveness of two dosing strategies, 10 mg compared to 20 mg three times daily for 28 days, on milk production in mothers of preterm infants [67]. Both dosing strategies showed breast milk volumes increasing with a clinically higher amount in the higher dosing approach, but the actual volumes were not given.

More recently, the EMPOWER trial by Asztalos et al. enrolled 90 mothers, who gave birth to preterm infants <30 weeks gestation, to receive domperidone 30 mg daily compared to a placebo for 14 days followed by all mothers receiving domperidone 30 mg daily for another 14 days [69]. More mothers achieved a 50% increase in milk volume after 14 days in the treated group (77.8%) compared to placebo (57.8%) (odds ratios 2.56; 95% confidence interval 1.02, 6.25; *p* = 0.04) ; however, the gain in actual volume was modest and not significantly different.

Each of the described studies evaluating domperidone as a means to augment breast milk production were significantly different in design and did not allow a more direct comparison. The studies were different in dosing approaches, timing and duration of treatment and the use or non-use of a placebo as well as outcome measures. The response to the interventions in the individual studies were different. Most, but not all, provided 24-h volumes as a measure for determining a response to domperidone. Two studies did not give actual values [64,67].

## 4. Clinical Efficacy

Overall, study findings indicate that metoclopramide is less efficacious than domperidone in augmenting breast milk production in mothers of preterm infants. Domperidone studies showed a modest increase in breast milk production but the approaches in dosing, timing and duration of treatment varied considerably in each trial. The cumulative dose in the trial by da Silva varied greatly compared to the trial by Knoppert [63,67]. In addition, even within a trial, mothers varied with respect to the cumulative dose [69]. Because the objectives of the individual studies varied, how breast milk volume was measured varied as well: 24-h volumes vs. percentages vs. volume per pump session.

Despite the varied approaches in outcome measures, the studies all demonstrated an increase in breast milk volume. However, it is important to note that 24-h volumes on average still remained below the target of 500 mL/day [63,65,66,68,69]. Recently, Grzeskowiak et al. conducted a meta-analysis which pooled five trials [63,65,68,69,70] which showed that short-term use of domperidone resulted in a modest 86 mL/day increase in expressed breast milk [71]. For a mother of a preterm infant weighing 1000 g and receiving enteral feeds at 160 mL/kg/day, this represents an opportunity to meet half of her infant’s feeds with her own breast milk, if not more, depending what her starting baseline volume had been. However, this modest volume increase may still fall short of the volume that an infant will need by term corrected age.

Whether there is a sustained effect on volume maintenance with galactogogues, and, in particular domperidone, is not clear. The EMPOWER study did follow the mothers to 6 weeks post term gestation. However, regardless of the assigned grouping, almost 60% of the study participants attempted to continue to provide breast milk and continued with some form of lactation inducing compounds at term gestation with the numbers dropping to just over 40% for the combined groups at 6 weeks post term gestation suggesting there was no sustained effect on breast milk production for the mothers in the trial [69]. At present, there are no studies that have looked at long-term use of domperidone beyond two or four weeks and whether it has an effect on sustained breast milk provision post initial hospital discharge.

## 5. Safety Issues

As noted earlier, metoclopromide exerts its effects centrally and can cause extrapyramidal side effects which may include tremor, bradykinesia and other dystonic reactions which are both dose and duration related [40,41]. These centrally-based side effects have prompted many clinicians to use domperidone rather than metoclopramide as their primary galactogogue. Domperidone’s use has grown exponentially for supporting mothers in breast milk production [72,73]. However, over the past decade, concerns have risen regarding the increased risk of prolongation of the Q-Tc interval, the risk of cardiac arrhythmias, and sudden cardiac death in the general adult population [74,75,76,77]. The relevance of these findings to women who are receiving this medication for lactation support is not clear and has been questioned [75]. However, given the wide use of domperidone to augment breast milk volumes, these concerns have led regulatory agencies, in particular the European Medicines Agency and Health Canada, to recommend caution in the use of domperidone and have provided dosing recommendations [78,79]. The most recent study to demonstrate these concerns, Smolina et al. identified 45,518 women from a provincial database who were dispensed domperidone in the first 6 months of their postpartum period [80]. Of these women, there were 21 women hospitalized for ventricular arrhythmia. The authors concluded that that there was a possible association between exposure to domperidone and hospitalization for ventricular arrhythmia (adjusted HR = 2.25, 95% CI 0.84–6.01), but that further research was needed to confirm this association. More recent studies have attempted to demonstrate an element of reassurance. In the EMPOWER trial, all of the 90 women enrolled had an ECG at study entry and at the end of the 4-week study period. Although not powered to detect a significant increase in cardiac arrhythmias, no women demonstrated any evidence of a QTc prolongation [69]. In addition, a recent review assessing QTc prolongation concluded that domperidone was not associated with QTc prolongation in healthy female volunteers [81]. A second major concern for safety related to domperidone is that of sudden cardiac death. Domperidone has been shown to have a 2.8-fold increased risk for sudden cardiac death in the general population [82,83]. However, no data to date demonstrates the risk specific to postpartum women. At present, recommendations from regulatory agencies suggest that a very small risk of cardiac arrhythmias and sudden cardiac death is associated with domperidone and clinician. If clinicians do prescribe domperidone for a non-authorized use such as lactation support, they should only use the established dosing guidelines of 30 mg daily [78,79].

As with any medication, there is always concern of transfer into breast milk. Lewis et al. evaluated the extent to which metoclopramide passed into breast milk in 10 mothers with full term infants. Maternal blood and milk samples collected 2 h after a single oral dose of 10 mg had plasma concentration of 69 ± 30 ng/mL and milk concentration 126 ± 42 ng/mL. The authors calculated that the average intake of metoclopramide by an infant would be less than 0.045 mg/kg/day which is well below the therapeutic doses used in preterm and term newborn infants [43]. Similarly, when evaluating 30 and 60 mg total daily doses of domperidone, the amounts transferred to breast milk were extremely low with median infant dose via milk being 0.04 and 0.07 µg/kg/day, respectively; this is far below the dose of 100–300 µg 3–4 times-a-day infants receive for gastrointestinal stasis [64]. With oral bioavailability at 15%, it is unlikely that pharmacologically meaningful amounts of domperidone reach the infant through the breast milk. No measures of infant serum concentrations of domperidone have been reported.

## 6. Summary

Following preterm delivery of an infant that is unable to breastfeed, measures should be in place to facilitate breast milk expression within one to six hours of birth as well as maintaining milk production [84,85]. The use of a galactogogue can be considered if additional support in breast milk production is needed especially in the presence of optimized pumping strategies. Although the trials demonstrate a modest efficacy in augmenting breast milk production at any point during the first 5 weeks of the postpartum period, earlier initiation by the end of the first week postpartum can be considered in order to optimize support for the mother. Based on the current literature and recommendations, domperidone, where available, should be the galactogogue of choice, with the dose of 10 mg three time daily for 14 days. There is inadequate evidence to guide treatment beyond 14 days. Careful history-taking and assessment are required to ensure domperidone is not administered to mothers at risk of cardiac arrhythmia. Mothers need to maintain pumping to facilitate the autocrine regulatory mechanism. Mothers should be assessed after 48–72 h of initiating domperidone to determine a response as evidence by an increase in breast milk volume.

## Figures and Tables

**Table 1:** Studies evaluating metoclopromide and breast milk production.

Study	Year	N	Placebo	Randomization	Intervention	Findings
Guzmán [42]	1979	21	Y	Y	20 mg TID 4 weeks	↑ BM, PRL
Lewis [43]	1980	20	Y	Y	10 mg TID 4 days	↑ BM
Tolino [44]	1981	10	N	N	10 mg TID 7 days	↑ BM, PRL
Kauppila [45]	1981	37	Y	Y	5–15 mg TID 2 weeks	↑ BM, PRL
Kauppila [46]	1981	17	N	N	10 mg TID 5 weeks	↑ BM, PRL
Kauppila [47]	1983	5	N	N	10 mg TID 5 days	↑ plasma levels in infant
de Gezelle [48]	1983	13	Y	Y	10 mg TID 8 days	↑ BM
Kauppila [49]	1985	24	Y	Y	10 mg TID 3 weeks	↑ BM
Gupta [50]	1985	32	N	N	10 mg TID	↑ lactation
Ehrenkranz [51]	1986	23	N	N	10 mg TID 7 days	↑ BM, basal PRL
Ertl [52]	1991	22	N	N	10 mg TID 5 days	↑ BM
Nemba [53]	1994	37	N	N	10 mg QID 5–11 days	↑ lactation
Toppare [54]	1994	60	N	N	10 mg TID	↑ lactation
Seema [55]	1997	50	N	N	10 mg TID 10 days	↑ lactation
Hansen [56]	2005	57	Y	Y	10 mg TID 10 days	No difference
Sakha [57]	2008	20	Y	Y	10 mg TID 8 days	No difference
Fife [58]	2011	19	Y	Y	10 mg TID 8 days	No difference

BM = breast milk; PRL = prolactin; TID = three times daily; Y = yes; N = no.

**Table 2:** Studies evaluating domperidone and breast milk production.

Study	Year	N	Placebo	Randomization	Intervention	Findings
De Leo [61]	1986	15	Y	N	10 mg TID 4 days	↑ lactation
Petraglia [62]	1985	17	Y	N	10 mg TID 10 days	↑ PRL, BM
da Silva [63]	2001	20	Y	Y	10 mg TID 7 days	↑ PRL, BM
Wan [64]	2008	6	N	Y	10 mg vs. 20 mg TID 1–2 weeks	↑ PRL, BM
Campbell-Yeo [65]	2010	46	Y	Y	10 mg TID 14 days	↑↑ PRL, BM
Ingram [66]	2012	80	N	Y	10 mg TID 10d or Metoclopramide 10 mg TID 10 days	↑ BM
Knoppert [67]	2013	15	N	Y	10 mg vs. 20 mg TID 4 weeks	↑ BM
Rai [68]	2016	32	Y	N	Unknown dose for 8 days	↑ BM
Asztalos [69]	2017	90	Y	Y	10 mg TID 14 days	↑ BM

BM = breast milk; PRL = prolactin; TID = three times daily; Y = yes; N = no.
